# Editorial for “Pre-Treatment T2-WI Based Radiomics Features for Prediction of Locally Advanced Rectal Cancer Non-Response to Neoadjuvant Chemoradiotherapy: A Preliminary Study”

**DOI:** 10.3390/cancers15030820

**Published:** 2023-01-29

**Authors:** Marta Zerunian, Andrea Laghi, Damiano Caruso

**Affiliations:** Radiology Unit, Department of Medical Surgical Sciences and Translational Medicine, Sapienza University of Rome—Sant’Andrea University Hospital, Via di Grottarossa, 1035-1039, 00189 Rome, Italy

Colorectal cancer still represents the third most frequent cancer in the world; around one-third of cancers are located in the rectum, with important differences in terms of diagnosis, treatment management, and survival compared to colon cancer [1].

Nowadays, MRI is the imaging technique for local staging; in cases of locally advanced rectal cancer, the standard of care includes neoadjuvant chemoradiotherapy (nCRT) with the goal of downstaging the tumoral mass by improving the possibility of performing a complete surgical resection. However, not all patients’ responses are similar, ranging from no response to a complete response to nCRT before treatment. The latter cases, which achieve a complete response to nCRT, undergo overtreatment with surgery, while non-responders might benefit from different treatment strategies without losing precious time. Despite several imaging biomarkers being present in the assessment of rectal cancer, at the moment an accurate one that is able to discriminate responders from non-responders has not been identified yet. Among the biomarkers of imaging with MRI, interesting results’ semantic features (T and N stage, circumferential resection margin, extramural venous invasion, and volume of the primary tumor at baseline) have been reached in functional MRI sequences such as diffusion-weighted imaging (DWI), dynamic contrast-enhanced (DCE) sequences, and radiomics [2,3,4]. In particular, radiomics is a post-processing technique applicable on every routinely imaging study acquired, able to extract a huge amount of ultrastructural data that characterize each pixel and that we are not able to discriminate with the naked eye. This technique, in fact, allows for the characterization of a certain tissue and or region/volume of interest in terms of the heterogeneity of the contiguous pixels with hundreds of quantitative mathematical and statistical descriptors, named radiomics features. This approach has been extensively applied to different tumoral lesions, enabling ultrastructural tissue characterization to seek non-invasive biomarkers able to improve tumor aggressiveness profiling, the response to therapy prediction, and survival in cancer patients [5,6]. Due to the huge amount of data and the relative novelty of the technique, standardization as well as validation are essential to clinical use, and this is the reason for the rapid increase in interest in the scientific community. All this potentiality also had a great impact on rectal cancer, with the main goal being to characterize the primary lesion with MRI to predict responses to treatment in a non-invasive manner before starting therapy [7]. 

This thrilling aspect has been investigated by Petresc B. and colleagues through an interesting paper entitled “Pre-Treatment T2-WI-Based Radiomics Features for the Prediction of Locally Advanced Rectal Cancer Non-Response to Neoadjuvant Chemoradiotherapy: A Preliminary Study”, published in the Special Issue “Radiomics and Cancers” of the 12th volume of the *Cancers* journal in 2020 [8]. This study has reached many readers and researchers, with a total of 23 citations at this time. 

The authors focused their analysis on a single non-contrast sequence, in opposition with other multiparametric MRI approaches [9]; the advantage of using the high-resolution axial oblique T2-weighted turbo spin echo (TSE) sequence only lies in the reduced presence of artifacts compared with other sequences (e.g., DWI), the presence of them in all MRI protocols for rectal cancer staging, and the lack of contrast medium injection.

A total of 67 patients with locally advanced rectal cancer were retrospectively enrolled and divided into test and validation; all of them underwent a baseline 1.5T MRI followed by nCRT and surgery with a consequent pathological report, considered as the reference standard. Each rectal lesion was carefully identified by two readers, and manual volumetric segmentation was performed by two readers independently to reduce biases. Radiomic features extraction was then performed with dedicated software, with and without the application of filters, for a total of 960 radiomics features. A scrupulous feature selection method was used by the authors to avoid overfitting and to select the most reproducible as well as significant features able to discriminate the responders from the non-responders group according to the pathological tumor regression grades (TRGs) proposed by Ryan R. et al. (TRGs 1–2 and 3, respectively) [10]. Seven features encountered these characteristics, and a radiomics score (rad-score) was built by weighting the features in accordance with LASSO coefficients. 

The rad-score of the training set showed brilliant results, with an area under the curve (AUC) of 0.94 in the prediction of a rectal cancer non-response to nCRT and an accuracy of 91%; the sensitivity, specificity, positive predictive value, and negative predictive values were 100%, 81% and 100%, respectively. 

Moreover, a complex model was tested by adding semantic variables with significant differences between the two groups, including in terms of tumor length, tumor differentiation grade, and mesorectal fascia status. In the complex model, the rad-score resulted in being an independent variable for the prediction of non-responses in LARC patients, with an odds ratio of more than six. Interestingly, the AUC of the semantic variables only reached 0.80, while it was raised to 0.97 in the complex score. 

Results were also confirmed in the validation set, with an AUC of 0.80 for the rad-score, a sensitivity of 75%, a specificity of 60%, a predictive positive value of 50%, and a negative predictive value of 75%. 

The authors underline how these results might impact the treatment choice at baseline examination, due to the possibility to discriminate responders from non-responders to nCRT before the beginning of it. 

Despite the study highlighting how radiomics might be a very powerful biomarker in the near future to assess responses to therapy at baseline before any treatment, a wider and multicenter validation of the results is auspicial to strengthening the results, with the aim of using this approach with a clinical scope soon. In fact, the implication of this imaging biomarker might pragmatically help clinicians and surgeons in treatment planning in a personalized view, with the goal of minimizing unnecessary treatments and improving survival. With the hope of stimulating curiosity in readers with this brief overview, the complete paper proposed by Petresc B. and colleagues is freely accessible through the Cancers journal website at https://www.mdpi.com/2072-6694/12/7/1894. 
